# An algorithm for tailoring pharmacotherapy for smoking cessation: results from a Delphi panel of international experts

**DOI:** 10.1136/tc.2008.025635

**Published:** 2008-10-13

**Authors:** P Bader, P McDonald, P Selby

**Affiliations:** 1 Consultants in Behavior Change, Toronto, Ontario; Ontario Tobacco Research Unit, Canada; 2 University of Waterloo; Population Health Research Group, Waterloo, Ontario; Ontario Tobacco Research Unit, Canada; 3 Departments of Family and Community Medicine and Psychiatry, Faculty of Medicine and the Dalla Lana School of Public Health, University of Toronto; Addictions Program, Centre for Addiction and Mental Health, Toronto, Ontario; Ontario Tobacco Research Unit, Canada

## Abstract

**Background:**

Evidence-based smoking cessation guidelines recommend nicotine replacement therapy (NRT), bupropion SR and varenicline as first-line therapy in combination with behavioural interventions. However, there are limited data to guide clinicians in recommending one form over another, using combinations, or matching individual smokers to particular forms.

**Objective:**

To develop decision rules for clinicians to guide differential prescribing practices and tailoring of pharmacotherapy for smoking cessation.

**Methods:**

A Delphi approach was used to build consensus among a panel of 37 international experts from various health disciplines. Through an iterative process, panellists responded to three rounds of questionnaires. Participants identified and ranked “best practices” used by them to tailor pharmacotherapy to aid smoking cessation. An independent panel of 10 experts provided cross-validation of findings.

**Results:**

There was a 100% response rate to all three rounds. A high level of consensus was achieved in determining the most important priorities: (1) factors to consider in prescribing pharmacotherapy: evidence, patient preference, patient experience; (2) combinations based on: failed attempt with monotherapy, patients with breakthrough cravings, level of tobacco dependence; (3) specific combinations, main categories: (a) two or more forms of NRT, (b) bupropion + form of NRT; (4) specific combinations, subcategories: (1a) patch + gum, (1b) patch + inhaler, (1c) patch + lozenge; (2a) bupropion + patch, (2b) bupropion + gum; (5) impact of comorbidities on selection of pharmacotherapy: contraindications, specific pharmacotherapy useful for certain comorbidities, dual purpose medications; (6) frequency of monitoring determined by patient needs and type of pharmacotherapy.

**Conclusion:**

An algorithm and guide were developed to assist clinicians in prescribing pharmacotherapy for smoking cessation. There appears to be good justification for “off-label” use such as higher doses of NRT or combination therapy in certain circumstances. This practical tool reflects best evidence to date of experts in tobacco cessation.

Helping smokers quit is a critical, yet often perplexing role for physicians. While pharmacotherapy generally doubles the odds of quitting successfully, these smoking cessation aids are not widely prescribed or used by smokers.^1^ ^2^ Although guidelines exist in several countries (United States, United Kingdom, France, Australia, New Zealand)^3^^–^8 that recommend nicotine replacement therapy (NRT) or bupropion SR as first-line medication, limited data are available to guide clinicians in selecting specific forms of pharmacotherapy for individual smokers. While varenicline, a new pharmacotherapeutic option, has demonstrated therapeutic superiority over existing first-line medications,^9^^–^12 post-marketing reviews have recently raised safety concerns regarding varenicline.^13^

The health benefits of smoking cessation are well documented. Smokers who quit reduce their risk of cardiovascular disease, lung disease, and cancer and increase their life expectancy substantially.^14^ While most smokers make several quit attempts before they succeed, about one in four who use any pharmacotherapy will eventually quit smoking.^15^ Evidence indicates that pharmacotherapy increases the odds of success and may reduce symptoms of withdrawal for those who smoke 10 or more cigarettes per day.^8^ ^15^ While a few studies have shown that pharmacotherapy works even in the absence of psychosocial therapies,^16^ ^17^ most studies show that combining pharmacotherapy and psychosocial treatments increases quit rates.^3^ ^8^

A Cochrane review^16^ including 123 trials concluded that all types of NRT increased the odds of quitting by approximately one-and-a-half to twofold. In addition, the effectiveness of NRT was independent of the intensity of behavioural support provided to the smoker. Bupropion SR and nortriptyline (antidepressants) were found to increase rates of smoking cessation in a Cochrane review of antidepressants including 53 trials.^18^ When prescribed as monotherapy, bupropion (31 trials) and nortriptyline (four trials) both doubled the odds of cessation. Bupropion and nortriptyline appear to have similar effectiveness to NRT. Other antidepressants (fluoxetine, sertraline, paroxetine, moclobemide, venlafaxine) have not shown significant benefit as an aid to smoking cessation.^18^ While studies of rimonabant have been completed,^19^ no reviews currently exist and there have been conflicting results regarding its efficacy in the US and Europe.^20^ ^21^

Clonidine (an α-adrenergic antagonist) was found to be an effective medication for smoking cessation, although findings were based on a small number of trials.^22^ Studies on other types of pharmacotherapy for smoking cessation are limited. Cochrane reviews have been conducted on anxiolytics,^23^ silver acetate,^24^ lobeline,^25^ mecamylamine^26^ and naltrexone,^27^ but findings are inconclusive owing to insufficient studies.

Varenicline, an α4 β2 nicotine receptor partial agonist, is the newest pharmacotherapy indicated for smoking cessation. It helps people to stop smoking by maintaining moderate levels of dopamine to counteract withdrawal symptoms and by reducing smoking satisfaction.^28^ Developed in 1997, it was approved in 2006 by the American Food and Drug Administration under the trade name Chantix, and by the European Medicines Evaluation Agency under the trade name Champix.

In the most recent Cochrane review, Cahill and colleagues^28^ found that varenicline “increased the chances of successful long-term smoking cessation between two- and threefold compared with pharmacologically unassisted quit attempts.” However, physicians prescribing varenicline need to be aware of the possible association with behavioural changes such as depressed mood, agitation and suicidal thoughts and behaviours. The US Food and Drug Administration recently issued a public health advisory, cautioning that there may be an increased risk of neuropsychiatric symptoms among patients taking varenicline.^13^ It is important to note that these symptoms may also arise as a result of smoking cessation with or without treatment, and causality has not yet been determined. The FDA is currently conducting a safety review. In particular, the safety and efficacy of varenicline in patients with psychiatric disorders is unknown (this population was excluded from pre-marketing trials). While an initial study in the UK (204 patients on NRT; 208 on varenicline) found no evidence that varenicline exacerbated mental illness,^29^ several individual case studies have been cited in the literature that report adverse psychiatric symptoms.^30^^–^34 The labelling of varenicline has now been changed to include warnings regarding adverse effects and stating that smokers considering use of varenicline should be screened for a history of psychiatric disorder and monitored closely for psychiatric adverse events.^35^ Clearly, “there is a need for individual community-based trials of varenicline, to test its efficacy and safety in smokers with varying comorbidities and risk patterns.”^28^

Although seven first-line types of pharmacotherapy (bupropion,nicotine gum, nicotine inhaler, nicotine lozenge, nicotine nasal spray, nicotine patch and varenicline) are recommended by the US Public Health Service Clinical Practice Guideline,^36^ there is no guidance on how to select a particular form of pharmacotherapy (or combination of pharmacotherapy) that will be most useful for individual patients. LeFoll and George^37^ report that “no clear threshold exists that can help clinicians decide whether a patient will benefit from a particular pharmacotherapy, and there is no consensus on which type of pharmacotherapy should be used first”. Few studies have been conducted combining different medications for increasing smoking cessation rates.^38^

Hughes and colleagues^39^ suggest that “studies simply showing that new treatments are more effective than placebo need to be supplemented by studies comparing treatments or testing patient-treatment matching theories”. Thus, clinicians are faced with the problem of selecting pharmacotherapy for patients with insufficient information and diverse opinions. They must rely solely on patient preference and past experience or on clinician familiarity with particular pharmacotherapy. This is a challenge that clinicians face on a daily basis.

When empirical evidence is inadequate, consensus methods can provide a vital component to synthesising knowledge. Consensus methods are particularly useful when dealing with conditions of uncertainty, insufficient data and incomplete theory.^40^ ^41^ A consensus method that has been widely used in health research is the Delphi approach. Delphi is a structured, systematic method using an iterative process to build consensus among a panel of experts.^42^ ^43^ It is a powerful tool for making the best use of less than perfect information. The Delphi consists of a multistage approach with each stage building on the results of the previous one. It allows for collecting and refining combined knowledge and experience from a group of experts from various disciplines. This approach has been used effectively by the investigators in a knowledge synthesis of smoking cessation among employed and unemployed young adults.^44^

The aim of this study was to develop decision rules (algorithm) for use by clinicians to guide prescribing practices of pharmacotherapy for smoking cessation. Experts in smoking cessation were recruited from 13 countries to participate in a Delphi consensus-building process. The results were synthesised into a clinical aid that can be widely used by clinicians who prescribe pharmacotherapy to smokers.

## METHODS

### Study design

A modified Delphi method was employed to identify and rank “best practices” used by healthcare practitioners in tailoring prescribing practices of pharmacotherapy to aid smoking cessation. Three Delphi rounds were conducted from May to September 2007, using primarily email communication. Thirty-seven panel members participated through an iterative process, responding anonymously to a series of questionnaires. Anonymity was intended to minimise “peer pressure” and enhance the free flow of ideas. Questions focused on how practitioners make decisions regarding selection of specific forms (or combination of forms) of pharmacotherapy for individual smokers. Also addressed was frequency of monitoring and follow-up, as well as the impact of medical or psychiatric comorbidities on pharmacotherapy selection.

### Expert panel

Experts were identified by an extensive search of the literature for their publications, keynote speakers on pharmacotherapy and smoking cessation at conferences, and by recommendations of the authors and other colleagues in the field. Seventy-three experts were invited to participate, with a 51% (37 out of 73) response rate. A deliberate effort was made to balance the panel with respect to gender, knowledge areas and geographic distribution. The panel comprised health practitioners (physicians, pharmacists) and researchers from 13 countries: 9 from Canada, 15 from the US and 13 from other countries including, Australia, Czech Republic, France, Greece, Netherlands, New Zealand, Norway, Russia, Spain, Sweden and Switzerland. Participants were involved in various types of practice: specialised smoking cessation, family practice, specialised practice (internal medicine, pulmonology), hospital-based (non-addiction or smoking cessation specific), addictions, mental health, academic (medical schools) and public health. Twenty-nine panellists were male; eight were female. Informed consent was obtained before the start of round 1. Participants were offered a small honorarium of $150 for completing the three rounds. Confidentiality of individual opinions of the panellists was maintained throughout the study (except to researchers). All findings represent the collective opinion of the panel. The research ethics board at the Centre for Addiction and Mental Health (CAMH) ruled that a formal review of the study protocol was not necessary, because the study participants were health experts and not at risk.

#### Round 1

The goal was to generate ideas and identify important priorities in prescribing or recommending pharmacotherapy for smoking cessation. Pilot testing of the questions was conducted with a small group of experts to ensure that the questions were clear. Panellists were asked to respond to four open-ended questions:

1. What factors do you consider in deciding which particular form of pharmacotherapy to prescribe or recommend?
2. Have you prescribed or recommended combinations of pharmacotherapy? If yes, for which type of patient? Describe.
3. What is the impact of medical or psychiatric comorbidities on your selection of pharmacotherapy?
4. What is your usual practice regarding frequency of monitoring patients while using pharmacotherapy?
5. In response to the first four questions, panellists generated a list of priorities and provided comments to support their suggestions.

#### Round 2

The goal was to identify priorities for each of the first four questions, with the content derived from the results of round 1. Content analysis was used to identify the major themes generated from the four central questions (and parts to questions) from round 1. Nine lists of priorities were produced and panellists were then asked to review and rank the items on each list. In addition, they were asked to provide reasons for their top choices in each category.

#### Round 3

The goal was to consolidate consensus. After scoring the responses from round 2, the top priorities were obtained for six key categories. A list of these priorities, as well as a brief summary of comments for each, was distributed to the panellists. The number of priorities for each category was chosen based on where there was a clear and distinct drop in priority scores. Consensus was consolidated as panellists were asked to either agree with the rankings or re-rank their choices for each category. They were asked to provide an explanation for any rankings with which they disagreed.

### Statistical analyses

Kendall’s W statistic and a principal components analysis (Q technique)^45^ ^46^ were computed, using SPSS software version 15, for the first two questions. This was not done on the remaining four questions because of the extremely high level of agreement.

Kendall’s W is a non-parametric statistical test that evaluates the level of agreement among raters with the overall ranking of items. This is a fairly conservative test regarding concordance because it considers the degree to which raters rank all priorities in a given question in exactly the same order. For example, in question 1 (factors considered in prescribing pharmacotherapy), Kendall’s W assesses the degree to which each rater ranked the seven priorities in identical order.

A principal components analysis (Q technique) was conducted on the correlation matrix among raters with each other. A very large first principal component (% variance) indicates that the raters are forming a single cluster (factor) or perspective, rather than distinct subgroups (factors) that would be evident if the variance was more distributed across the first few principal components.

## RESULTS

All 37 members (100%) of the panel completed the entire process. After three Delphi rounds, the panel members reached a high level of consensus in determining the most important priorities for prescribing and recommending pharmacotherapy for smoking cessation.

### Round 1

Responses from four open-ended questions posed in round 1 were analysed and categorised according to common themes to generate nine lists of priorities (appendix I available from corresponding author). Explanations and examples given by panellists were also summarised and included.

### Round 2

The nine lists of priorities (together with a summary of comments) were sent to panel members. They were asked to rank all items on each list and to provide reasons for their top choices. Then, their rankings were combined to produce a total score for each priority (for example, for a list of 10 items, the first or highest ranked item  =  10 points, second = 9 points … 10th = 1 point). The results are given in appendix II (available from corresponding author). The number of priorities was based on where there was a clear and distinct drop in score.

### Round 3

In this final consensus-building round, scores were calculated to generate priorities for the six questions. These priorities, and a summary of comments for each, were sent to panel members who were asked to either agree or disagree with the rankings. They were also requested to provide explanations for any disagreement with a ranking. Comments provided by panellists during all three rounds were analysed for content and major themes.

The results in table 1 show a high degree of consensus on the most important priorities for prescribing pharmacotherapy for smoking cessation. Percentage of agreement was calculated by summing the number of raters who agreed with the factor divided by the total number of raters (x 100). The average agreement with the six questions was 84%. Agreement with specific items in each category, ranged from 73% to 97%.

**Table 1:** Level of agreement by question and priority item

Priority No	Questions and priority items	Agreement	
		Number out of 37	%
	**1. Factors considered in prescribing pharmacotherapy(ies)**		
1	Evidence	32	86
2	Patient preference	30	81
3	Patient experience	29	78
4	Patient needs	28	76
5	Patient history	27	73
6	Patient’s clinical suitability	28	76
7	Potential drug interactions/side effects	28	76
	Mean	29	78
	Kendall’s W (coefficient of concordance)	0.68	
	Statistical significance	p<0.001	
	First principal component	79.4% of variance	
	**2. Combinations of pharmacotherapies prescribed based on**		
1	Failed attempt with monotherapy	30	81
2	Patients with breakthrough cravings	30	81
3	Level of dependence	27	73
4	Multiple failed attempts	31	84
5	Patients with nicotine withdrawal	31	84
	Mean	29	78
	Kendall’s W (coefficient of concordance)	0.70	
	Statistical significance	p<0.001	
	First principal component	78.6% of variance	
	**3. Specific combinations of pharmacotherapies: main categories**		
1	Two or more forms of NRT	34	92
2	Bupropion + form of NRT	34	92
	Mean	34	92
	**4. Specific combinations of pharmacotherapies: subcategories**		
	*2 or more forms of NRT*		
1	Patch + gum	34	92
2	Patch + inhaler	32	86
3	Patch + lozenge	32	86
	Mean	33	88
	*Bupropion + form of NRT*		
1	Bupropion + patch	33	89
2	Bupropion + gum	33	89
	Mean	33	89
	**5. Impact of comorbities on the selection of pharmacotherapy(ies)**		
1	Contraindications	36	97
2	Specific pharmacotherapies useful for certain comorbidities	36	97
3	Dual purpose medications	36	97
	Mean	36	97
	**6. Frequency of monitoring determined by**		
1	Patient needs	36	97
2	Type of pharmacotherapy	36	97
	Mean	36	97

### Question 1: Factors considered in prescribing pharmacotherapy

Kendall’s W coefficient of concordance was 0.68, which was statistically significant at p<0.001. The high level of concordance was further evidenced by the first principal component (79.3%), accounting for almost 80% of the variance among raters. This underscores that the raters largely agreed on the ranking of items for question 1. The highest levels of agreement are with the top three items: (1) evidence, 86%; (2) patient preference, 81%; and (3) patient experience, 78%.

### Question 2: Combinations of pharmacotherapy

Kendall’s W coefficient of concordance was 0.70, which was statistically significant at p<0.001. The high level of concordance was further evidenced by the first principal component accounting for 78.6% of the variance among raters. Interestingly, the highest levels of agreement were with the lower ranked items, (4) and (5): (4) Multiple failed attempts, 84%; (5) Patients with nicotine withdrawal, 84%. Agreement with the first two items was also high: (1) Failed attempt with monotherapy, 81%; (2) Patients with breakthrough cravings, 81%. There was least agreement on the middle ranked item (3) Level of dependence, at 73%.

### Question 3: Specific combinations of pharmacotherapy, main categories

Overall level of agreement of priorities for specific pharmacotherapies was particularly high at 92%.

### Question 4: Specific combinations of pharmacotherapy, subcategories

There was high overall agreement (81%) of the priorities for subcategories of pharmacotherapies under the main categories identified in question 3. The highest level of agreement was item (1) under two or more forms of NRT, patch + gum, at 92%. Bupropion + patch and bupropion + gum were also compelling at 89%.

### Question 5: Impact of comorbidities

Ninety-five per cent (36 out of 37 panelists) agreed with the specific rankings for this question.

### Question 6: Frequency of monitoring

Overall agreement (97%) was significantly high for this question—only one panelist disagreed with the ranking of items.

An important addition to this study was an independent panel of 10 international experts (two from Canada, three from the US, two from the UK, one from Denmark, one from France and one from Poland) to provide external validation of our findings. The final rankings of the original panel were sent to the independent panel by email, at the completion of round 3. They were asked to agree or disagree with the rankings for the six categories. Also, they were asked to re-rank any priorities with which they disagreed and to provide an explanation for their choice.

The level of agreement with priority rankings for each question between the independent panel and the original panel was quantified. The results of the independent panel rankings showed a high degree of congruence with the rankings of the original Delphi panel. The average agreement with the six questions was 87%. Agreement with specific items in each category, ranged from 70% to 100%. For question 1, the level of agreement was 74%; question 2, 74%; question 3, 100%; question 4, 84%, question 5, 100% and question 6, 90%.

### Algorithm

**Figure 1:**
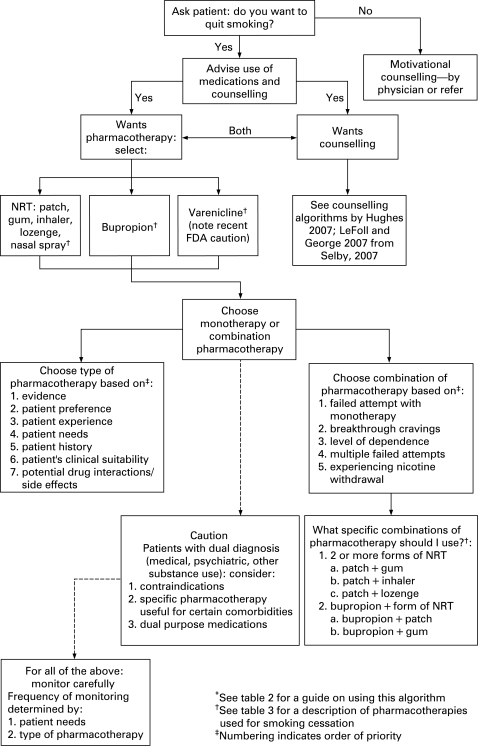
Algorithm for tailoring pharmacotherapy for smoking cessation*†

An algorithm to guide decision-making regarding pharmacotherapy for smoking cessation is given in figure 1. This algorithm integrates key findings from our study. Also, it complements and extends algorithms developed by Selby^47^ and published in a paper by LeFoll and George^37^ and the one developed by Hughes,^48^ by specifying how to select a specific type or combination of pharmacotherapy (fig 1). A guide for using the algorithm was developed from the comments of the panel members (table 2). Table 3 provides a description of types of pharmacotherapy used for smoking cessation.

**Table 2:** Guide for using the algorithm in figure 1 (*quotations included are from Delphi panel participants)

**Factors to consider in prescribing pharmacotherapy**
Three distinct types of pharmacotherapy have demonstrated efficacy for smoking cessation: (a) nicotine replacement therapy (including patch, gum, inhaler, lozenge, nasal spray), (b) bupropion and (c) varenicline (for a description of these pharmacotherapy, including dose and side effects/drug interactions, see table 3). Selecting a particular type of pharmacotherapy should be guided by the following seven factors:
*1. Evidence*
The importance of evidence-based medicine is the top priority in considering which form of pharmacotherapy to prescribe or recommend to a patient. The decision to prescribe smoking cessation medications needs to be based on evidence of effectiveness and safety (see Fiore *et al*^36^).
*2. Patient preference*
Patient preference is an important priority in facilitating adherence to the treatment protocol. There is no value in prescribing or recommending a medication that a patient will not take. “It is essential that the patient be comfortable with the decision, have reasonable expectations for product efficacy, and have confidence in their ability to use the medication appropriately”. Preference is particularly important if a patient does not want to use a specific product. However, patient preference can be modified through an informed and shared decision-making process between the clinician and patient.
*3. Patient experience*
The patient’s expectation of success is exceedingly important in determining actual success. Expectations are often informed by experience. Therefore, a patient’s experience with smoking cessation attempts and use of pharmacotherapy needs to be a significant factor in influencing choice of pharmacotherapy. “A clinician must understand what the patient has tried and why the patient did not succeed”. If the patient was successful with a particular medication for a period of time, it may be prudent to try the same medication again; if unsuccessful with a particular medication, then probably should not use again.
*4. Patient needs*
Because there is little evidence-based information to guide tailoring of specific pharmacotherapy to specific patients, patient needs are vital. Consideration of patient needs is important in determining their willingness to use medications, the ease of use of various smoking cessation products and likelihood of compliance. Other patient needs to take into account before prescribing or recommending a particular pharmacotherapy include: extent and severity of cravings, situations or times when cravings are strongest, triggers for smoking, specific hurdles to overcome, etc.
*5. Patient history*
“Patient history provides the framework within which I can prescribe”. Many patients have comorbidities (medical, psychiatric, alcohol/drug abuse) which need to be taken into account. For example, a patient with a history of alcohol abuse or seizures would be excluded from bupropion use. Smoking history, past quit attempts and experience with pharmacotherapy are all factors influencing the decision of pharmacotherapy choice.
*6. Patient clinical suitability for pharmacotherapy*
Some patients may not be suitable for pharmacotherapy interventions and potential contraindications need to be considered. Generally, pharmacotherapy would not be recommended for patients having a low level of nicotine dependence. In addition, a patient may prefer a non-pharmacological approach to treatment.
*7. Potential drug interactions/side effects*
Issues of safety are fundamental in determining choice of pharmacotherapy. Contraindications, use of other medications, and the side effect profile all need to be considered. However, this is generally a minor problem with cessation drugs. “Potential drug interactions are a show-stopper when it is relevant, but it is rarely an issue, so it is important but infrequent”.
**Combinations of pharmacotherapy**
For some patients, choosing a combination of pharmacotherapy will increase their ability to stop smoking. Combination pharmacotherapy is indicated for patients based on five factors:
*1. Failed attempt with monotherapy*
Use of monotherapy which resulted in a failure to quit smoking is the top priority when considering use of combination pharmacotherapy. The general principle is that intensity of medications should be increased when monotherapy has resulted in relapse. A caveat is that the medication was used appropriately and that there was “a ‘true’ attempt to quit”.
*2. Patients with breakthrough cravings*
Breakthrough cravings may be an indication that more treatment is needed. An additional form of NRT or an addition of NRT (as needed) to a non-NRT oral medication may be helpful. Combinations of NRT can be used for steady-state delivery (patch) and as needed (gum/lozenge).
*3. Level of dependence*
Highly dependent smokers are more likely to benefit from combination pharmacotherapy. It may be important to begin with combination pharmacotherapy for these individuals. Because this group has a difficult time in quitting smoking, combination therapy may facilitate increased success.
*4. Multiple failed attempts*
Multiple failed attempts may be an indication that more intensive therapy is needed. “Careful assessment of previous attempts usually reveals complex situations which are more likely to be addressed with combination pharmacotherapy.” However, it is important to keep in mind that failed attempts may also be based on patient lack of commitment rather than insufficient medication.
*5. Patients with nicotine withdrawal*
Patients experiencing nicotine withdrawal can be a trigger for their relapse to smoking. The combination of pharmacotherapies (for example, addition of NRT to another pharmacotherapy) can be a helpful response for managing nicotine withdrawal symptoms.
**Specific combinations of pharmacotherapy**
When prescribing or recommending combinations of pharmacotherapy, first select combinations of NRT. Then, prescribe a combination of bupropion and NRT for more heavily dependent patients.
*1. Two more forms of NRT*
The use of two or more forms of NRT has the strongest evidence base and is the most commonly used form of combination therapy. There is a high level of confidence that this combination can be used safely and effectively. “This approach permits optimal titration of NRT to meet nicotine needs and can be achieved easily and cheaply”.
*2. Bupropion + form of NRT*
Bupropion plus a form of NRT can be effective for some patients. This combination is generally used in more heavily dependent patients.
**Impact of comorbidities on selection of pharmacotherapy**
When prescribing pharmacotherapy to patients having a dual diagnosis (that is, medical, psychiatric or other substance use in addition to smoking), specific attention should be given to:
*1. Contraindications*
Attention to contraindications is the top priority in the selection of type of pharmacotherapy in patients with comorbidities. Ensuring the safety of a patient is always of primary importance in prescribing or recommending medications. Contraindications are primarily an issue with use of bupropion (that is, history of seizures, alcohol problems) and with patients who are already taking other medications.
*2. Specific pharmacotherapy useful for certain comorbidities*
Specific pharmacotherapy may be useful for treatment of certain comorbidities in addition to smoking cessation. For example, bupropion may be a good choice for depressed patients who want to quit smoking. However, for patients with anxiety disorders or eating disorders, bupropion would not be a good choice.
*3. Dual purpose medications*
“It’s nice to treat two things with one med so if I can do that I will”. Most common is use of bupropion for depressed patients who want to quit smoking. Bupropion can also be useful for patients who do not want to gain weight. Dual purpose medications may have added value in enhancing compliance.
**Frequency of monitoring**
All patients taking pharmacotherapy should be monitored carefully. The frequency of monitoring should be determined by:
*1. Patient need*
The top priority for frequency of monitoring should be determined by patient needs. For example, patients with multiple or difficult quit attempts will likely require more support.
*2. Type of pharmacotherapy*
Some types of pharmacotherapy may require more frequent monitoring, particularly if there is potential for adverse events (for example, drug interaction, side effects).

**Table 3:** Pharmacotherapy used for smoking cessation^36^ ^47^

Drug	Dose	Side effects/drug interactions	Comments
**NRT: sustained release**Nicotine transdermal patch (Habitrol, Nicoderm, generics)	>20 cigarettes/day: 1 patch (21 mg/24 h) for 4–6 weeks, then taper to 14 mg/day for 2–4 weeks, then 7 mg per day for 2–4 weeks.If patient has cardiovascular disease, weighs less than 45 kg or smokes <½ pack/day begin with 14 mg/24 h×6 weeks then ↓ to 7 mg/24 h × 2 weeksNB: 16-h patches are available in some countries	**Side effects:**Skin sensitivity and irritation (most common); abnormal dreams; insomnia; nausea, dyspepsia	Start patch on quit date. Advise not to smoke cigarettes while using the patch, though this is generally safe and does not indicate treatment failure. Educate users on the signs and symptoms of nicotine toxicity
**NRT: immediate release**Nicorette inhaler (nicotine inhaler)	Available in 4 mg strength.Encourage patient to use at least six doses/day for the ?rst 3–6 weeks.Max 12/day.Tapering: gradual reduction in use over next 6–12 weeks, stopping when reduced to 1–2/day	**Side effects:**Mild local irritation of mouth and throat, coughing, rhinitis that may decline with continued use	Not a true inhaler—the nicotine is delivered and absorbed buccally.“Hand-mouth” activity from using the inhaler is preferred by some quitters while others ?nd it to be a trigger. Useful in those with poor oral health or dentures and in those who cannot chew gum
**NRT: immediate release**Nicotine polacrilex gum (Nicorette Gum)	10–12 pieces per day initially (2 mg or 4 mg pieces) to maximum of 20 pieces per day, for 12 weeks.Tapering: 1 piece/day each week, as withdrawal symptoms allow	**Side effects:**Mouth soreness, hiccups, dyspepsia, jaw ache	Use 4 mg in heavily dependent smokers. May be used for temporary abstinence—eg, to comply with smoking restrictions on aeroplanes
**NRT: immediate release**Nicotine lozenge	1 lozenge (2 mg or 4 mg lozenges) every 1–2 h up to 6 weeks; weeks 7–9, every 2–4 h; weeks 10–12, every 4–8 h	**Side effects:**Nausea, hiccups, heartburn, headache, coughing	
**NRT: immediate release**Nicotine nasal spray	1.0 mg of nicotine per spray (10-ml bottle contains 100 mg nicotine) 1–2 doses/h up to 40 doses per day; for 3 months	**Side effects:**Mild nasal/throat irritation	
**Antidepressant:**Bupropion (Zyban, generics)	150 mg daily × 3 days then 150 mg twice daily × 7–12 weeks. Begin 1–2 weeks before the selected quit date	**Side effects:**Insomnia, dry mouth**Drug interactions:**Clearance of bupropion may be ↓ by inhibitors (for example, ticlopidine) or ↑ by inducers (for example, phenobarbital, phenytoin, primidone) of CYP2B6. May ↓ clearance of other substrates of CYP2B6 (for example, cyclophosphamide, ketamine, promethazine, propofol, selegiline). MAOIs, levodopa, amantadine may ↑ toxicity. May be safely combined with NRT (monitor for treatment-emergent hypertension)	Not recommended in patients with conditions predisposing to seizures, history of seizures, current eating disorder or severe hepatic impairment.Least expensive of oral medications indicated for smoking cessation
**Nicotine receptor partial agonists**Varenicline (Champix, Chantix)	0.5 mg daily for 3 days, then twice daily for 4 days then 1 mg by mouth twice daily for 12 weeks.Patient should quit smoking 1–2 weeks after starting the medication. Reassess if patient is still smoking 4 weeks after starting medication; can be continued for an additional 12 weeks if patient has bene?ted. No tapering necessary	Side effects: nausea, sleep disturbance, abnormal/vivid/strange dreams.Drug interactions: should not be combined with NRT therapy because of increased risk of adverse effects	Does not induce cytochrome P450 enzymes; excreted renally unchanged.Smokers considering use of varenicline should be screened for a history of psychiatric disorders, have close monitoring, and be advised to report any adverse effects they might experience. Care and close surveillance needs to be taken if prescribing to patients with psychiatric disorders

## DISCUSSION

The Delphi, based on well-researched principles, is a widely accepted method used in studies ranging from technology forecasting to drug abuse.^49^ It is a powerful tool for making the best use of less than perfect information. It should be noted that the existence of consensus from a Delphi process does not mean that the “correct” answer has been found. However, this process does aim to “negotiate a reality that can then be useful in moving a particular field forward, planning for the future or even changing the future by forecasting its events.”^50^

The consensus view in this study offers practical and realistic guidance to clinicians. It reflects the best evidence to date of expert views in the tobacco cessation field. Participants were enthusiastic about the topic, evidenced by 100% participation in all three rounds. Achieving a 100% response rate is rare in a Delphi process. This method asks much more of respondents than a single survey and the potential for low response rates increases exponentially with each round. This high response rate was the result of the participants’ fervent interest in the topic and the investigators’ strategy to improve follow-up. The use of an independent panel is an innovative addition to standard Delphi methodology. The high level of agreement of the independent panel with the consensus achieved by the original panel strengthens the credibility of our findings.

Cost of smoking cessation medications can be a key concern since all available pharmacotherapy treatments are relatively expensive. However, cost varies across countries, from free provision to insurance coverage to full payment required by patients. While cost was listed as a concern by experts in round 1 of the Delphi process, it did not emerge as a top priority from the clinician’s perspective. If focus groups had been conducted with smokers, it may have emerged as a greater priority.^44^

The algorithm developed from this study (fig 1) provides a practical tool for clinicians. This reflects the current approach of experts in smoking cessation and may serve as a guide for practitioners. It also adds value to current knowledge and existing algorithms^37^ ^47^ ^48^ by prioritising factors to consider in prescribing pharmacotherapy. The expert comments summarised in table 2 serve as a guide for training and using the algorithm.

A major strength of this study is the international panel. However, a limitation in that not all panellists have equal access to the range of pharmacotherapy reviewed. At the time of this study, varenicline was just being introduced into many jurisdictions. Therefore, experts may not have been as familiar with it. Given the limited availability of varenicline and recent safety concerns, this algorithm may need to be updated in the future. Although varenicline has demonstrated clear evidence of efficacy in smoking cessation, it is essential to consider the safety concerns and risks recently raised by the FDA. Smokers considering use of varenicline should be screened for a history of psychiatric disorders, have close monitoring, and be advised to report any adverse effects they might experience. Particular care and monitoring need to be taken if prescribing to patients with psychiatric disorders.

There was not an exhaustive representation of experts by countries. Although desirable, this was beyond the scope and means of this study. Data were collected from the Delphi panel members regarding potential conflicts of interest. Twenty-four of the 37 panellists indicated that they have received some funding from the pharmaceutical industry (details are available from the corresponding author). However, panel members with potential conflicts of interest did not respond differently from those who had no conflicts. Therefore, this did not appear to affect our recommendations.

## CONCLUSION

The Delphi panel of 37 experts clearly recognised the wide gap in the smoking cessation literature regarding effective prescribing practices. Our study addressed this gap by achieving a high level of consensus on priorities in prescribing pharmacotherapy. The validation by an independent expert group provides further assurance. The algorithm (fig 1), guide (table 2) and pharmacotherapy description (table 3) can assist healthcare practitioners in the art of prescribing pharmacotherapy for smoking cessation. These clinical aids will hopefully engage and assist a larger number of clinicians to incorporate smoking cessation interventions in their practice.

A defining characteristic of tobacco control—most likely a necessary one—is that action has typically preceded a strong body of research understanding. Knowledge has derived from analysis of that experience.^51^
